# Dynamic clade transitions and the influence of vaccine rollout on the spatiotemporal circulation of SARS-CoV-2 variants in São Paulo, Brazil

**DOI:** 10.21203/rs.3.rs-3788142/v1

**Published:** 2024-01-22

**Authors:** Cecília Artico Banho, Beatriz de Carvalho Marques, Lívia Sacchetto, Ana Karoline Sepedro Lima, Maisa Carla Pereira Parra, Alex Ranieri Jeronimo Lima, Gabriela Ribeiro, Antonio Jorge Martins, Claudia Renata dos Santos Barros, Maria Carolina Elias, Sandra Coccuzzo Sampaio, Svetoslav Nanev Slavov, Evandra Strazza Rodrigues, Elaine Vieira Santos, Dimas Tadeu Covas, Simone Kashima, Ricardo Augusto Brassaloti, Bruna Petry, Luan Gaspar Clemente, Luiz Lehmann Coutinho, Patricia Akemi Assato, Felipe Allan da Silva da Costa, Jayme A. Souza-Neto, Rejane Maria Tommasini Grotto, Mirele Daiana Poleti, Jessika Cristina Chagas Lesbon, Elisangela Chicaroni Mattos, Heidge Fukumasu, Marta Giovanetti, Luiz Carlos Junior Alcantara, Paula Rahal, João Fernando Pessoa Araújo, Benjamin M. Althouse, Nikos Vasilakis, Maurício Lacerda Nogueira

**Affiliations:** 1 Laboratório de Pesquisas em Virologia, Faculdade de Medicina de São José do Rio Preto; São José do Rio Preto, São Paulo, Brazil; 2 Center for Viral Surveillance and Serological Assessment (CeVIVAS), Butantan Institute, São Paulo, Brazil; 3 University of São Paulo, Ribeirão Preto Medical School, Blood Center of Ribeirão Preto, Ribeirão Preto, SP, Brazil; 4 University of São Paulo, Centro de Genômica Funcional da ESALQ, Piracicaba, SP, Brazil; 5 São Paulo State University (UNESP), School of Agricultural Sciences, Department of Bioprocesses and Biotechnology, Botucatu, Brazil; 6 São Paulo State University (UNESP), School of Agricultural Sciences, Botucatu, Brazil; 7 Molecular Biology Laboratory, Applied Biotechnology Laboratory, Clinical Hospital of the Botucatu Medical School, Brazil; 8 Department of Veterinary Medicine, School of Animal Science and Food Engineering, University of São Paulo, Pirassununga, São Paulo, Brazil; 9 Oswaldo Cruz Foundation, FIOCRUZ, Rio de Janeiro, Brazil; 10 Climate Amplified Diseases And Epidemics (CLIMADE), Brazil, Americas; 11 Sciences and Technologies for Sustainable Development and One Health, Universita Campus Bio-Medico di Roma, Italy; 12 Laboratório de Estudos Genômicos, Departamento de Biologia, Instituto de Biociências Letras e Ciências Exatas (IBILCE), Universidade Estadual Paulista (Unesp), São José do Rio Preto, Brazil; 13 Instituto de Biotecnologia, Universidade Estadual Paulista (Unesp), Botucatu, Brazil; 14 Laboratório de Microbiologia Molecular, Instituto de Ciências da Saúde, Universidade Feevale, Novo Hamburgo, Brazil; 15 Department of Biology, New Mexico State University, Las Cruces, NM; 16 Information School, University of Washington, Seattle, WA; 17 Department of Pathology, University of Texas Medical Branch; Galveston, Texas, United States of America; 18 Center for Vector-Borne and Zoonotic Diseases, University of Texas Medical Branch, Galveston, Texas, United States of America; 19 Institute for Human Infection and Immunity, University of Texas Medical Branch, Galveston, Texas, United States of America

**Keywords:** COVID-19, Genomic surveillance, Variant of Concern, Vaccine effectiveness, Epidemiology

## Abstract

Since 2021, the emergence of variants of concern (VOC) has led Brazil to experience record numbers of in COVID-19 cases and deaths. The expanded spread of the SARS-CoV-2 combined with a low vaccination rate has contributed to the emergence of new mutations that may enhance viral fitness, leading to the persistence of the disease. Due to limitations in the real-time genomic monitoring of new variants in some Brazilian states, we aimed to investigate whether genomic surveillance, coupled with epidemiological data and SARS-CoV-2 variants spatiotemporal spread in a smaller region, can reflect the pandemic progression at a national level. Our findings revealed three SARS-CoV-2 variant replacements from 2021 to early 2022, corresponding to the introduction and increase in the frequency of Gamma, Delta, and Omicron variants, as indicated by peaks of the Effective Reproductive Number (Reff). These distinct clade replacements triggered two waves of COVID-19 cases, influenced by the increasing vaccine uptake over time. Our results indicated that the effectiveness of vaccination in preventing new cases during the Delta and Omicron circulations was six and eleven times higher, respectively, than during the period when Gamma was predominant, and it was highly efficient in reducing the number of deaths. Furthermore, we demonstrated that genomic monitoring at a local level can reflect the national trends in the spread and evolution of SARS-CoV-2.

## Introduction

Several SARS-CoV-2 variants have emerged over the course of the COVID-19 pandemic, driven by an unprecedented transmission rate and the rapid evolution of RNA virus genomes ^1–3^. Lineages of SARS-CoV-2 harboring mutations associated with increased transmissibility, pathogenesis, and immune evasion began to appear in different countries in mid-2020. These emerging lineages have been classified by the World Health Organization (WHO) as variants of concern (VOC): Alpha ^7^, Beta ^8^, Gamma ^9^, Delta ^10^, and Omicron^11^. These VOCs have replaced circulating strains, leading to subsequent waves of COVID-19 infection and healthcare system overloads, and the need of frequent vaccine updates ^4–6,9^. The increasing number of cases from these new waves has reinforced the need for continuous genomic surveillance of SARS-CoV-2 ^12–14^. Studies on the epidemiology and the spatiotemporal history of SARS-CoV-2 transmission can effectively contribute to implementing real-time public health measures to contain the viral spread. While WHO has classified SARS-CoV-2 transmission and its disease as a continuous health problem, a robust genomic surveillance, variant identification, and the evaluation of their pathogenic potential, are fundamental elements for policymaking and disease containment^3,5^.

Brazil was an epicenter of the COVID-19 pandemic as early as March 2020, and by December 2023, it had recorded 38,106,633 cases and 708,021 deaths^15^. Resources for real-time genomic surveillance are limited in most Brazilian states, which makes identifying the emergence of regional variants or mutations challenging in a country the size of Brazil. One strategy to address this challenge is to monitor the lineages that circulate in a smaller region, such as a midsize city, which can reflect the Brazilian epidemiological landscape. São José do Rio Preto (SJdRP) is a suitable location for such monitoring, as the city presented the third highest number of COVID-19 cases and deaths in the state of São Paulo (SP) ^16^. Furthermore, it housed one of the country’s largest and most significant hospital complexes, the Hospital de Base de São José do Rio Preto (HB). This reference center, serving more than two million inhabitants, was at the forefront of COVID-19 care and treatment for the state ^17^ and, with its constant exchange of SARS-CoV-2 variants, serves as an important model for visualizing and predicting viral transmission patterns. Here, we investigated how the introduction and spread of different SARS-CoV-2 VOCs combined with vaccination rollout have shaped the progression of COVID-19 in a single health district in northwestern São Paulo state.

## Results

### SARS-CoV-2 variant report

**Figure 1.**
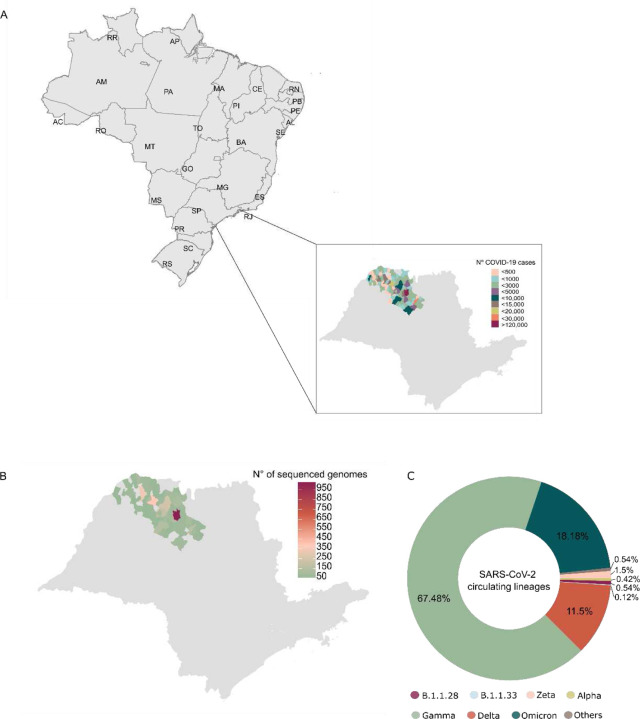
SARS-CoV-2 lineages genomic survaillance in northwestern of São Paulo state. A) Map of Brazil showing the number of COVID-19 cases reported in the municipalities from the 15^th^ Regional Health District (RHD XV) in northwestern São Paulo state from January 2021 to April 2022. B) Map of São Paulo state showing the number of sequenced genomes obtained from the municipalities from the RHD XV from January 2021 to April 2022. C) Prevalence of SARS-CoV-2 lineages detected in the municipalities from the RHD XV from January 2021 to April 2022 by genomic surveillance.

São Paulo state recorded the highest numbers of COVID-19 cases and deaths in Brazil ^15,16^; in response to that, the São Paulo State Network for Pandemic Alert of Emerging SARS-CoV-2 Variants (SPNPAESV) was implemented in early 2021 to monitor the real-time evolution of circulating VOC and variants of interest (VOI) within the state. São Paulo state is divided into 17 regional health districts by the State Secretary of Health (SES-SP), facilitating the planning and articulation of health policies in alignment with the Brazilian national public health system (*Sistema Único de Saúde*, SUS) ^18^. Owing to substantial efforts from various research centers across each regional district, the SPNPAESV successfully sequenced 3,306 complete SARS-CoV-2 genomes from the Regional Health District (RHD) XV encompassing 102 cities, including SJdRP in the northwestern part of São Paulo state (Figure 1A).

In our study, SARS-CoV-2 sequences from 85 cities were included (Figure 1B); sequences from SJdRP were the most representative, accounting for 29.3% of the sequenced genomes, and a total of 14 SARS-CoV-2 lineages were detected from January 2021 to April 2022. Of these lineages, the VOC comprised most of the sequenced genomes, with Gamma being the most prevalent (n=2,227, 67.48%), followed by Omicron (n=600, 18.18%) and Delta (n=381, 11.5%). Interestingly, the circulation of the Alpha variant (n=1, 0.42%) was limited in the sampled municipalities. Other lineages, mostly detected in early 2021, were present at a low frequencies, including Zeta (n=38, 1.15%), B.1.1.28 (n=18, 0.54%), B.1.1.33 (n=4, 0.12%), and others such as B.1, B.1.1, B.1.2, B.1.1.332, B.1.1.393, N.9, and P.4 (n=18, 0.54%) (Figure 1C, Supplementary Table 1).

**Figure 2.**
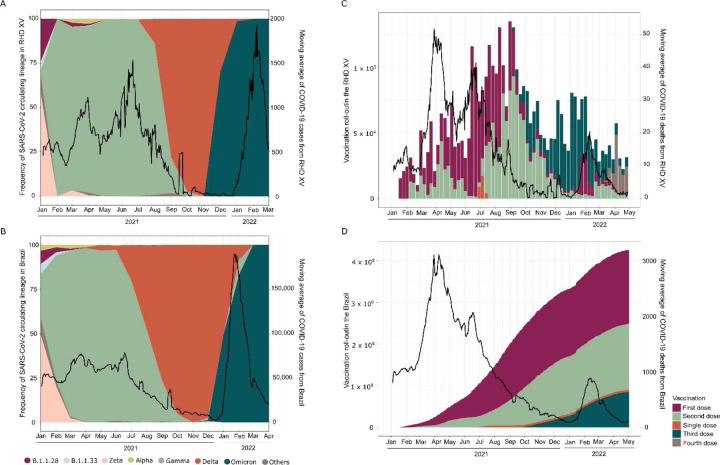
Genomic epidemiology of SARS-CoV-2 epidemics in northwestern São Paulo state. Proportion of SARS-CoV-2 lineages circulating over time and associations with the moving average of COVID-19 cases reported in municipalities from the 15^th^ regional Health District (RHD XV) (A) and Brazil (B); Vaccination coverage and its effect on the number of deaths reported in the RHD XV (C) and Brazil (D).

Analyzing the distribution of SARS-CoV-2 lineages over time in the municipalities within the RHD XV, we observed that Zeta was the prevalent VOI in January 2021 (58.32%) and co-circulated with the other five variants (Supplementary Table 1). The introduction of Gamma was detected in late January; from then on, we observed the replacement of nearly all the other circulating SARS-CoV-2 variants, along with a sharp increase in COVID-19 case numbers (Figure 2A). Consequently, from March to September 2021, the VOC Gamma (P.1 and its sub-lineages) was the predominant lineage within the RHD XV. We identified the introduction of the Delta VOC in early August 2021 (in the city of Jales). This variant rapidly increased in frequency, replacing the Gamma variant by October of that year. Delta remained the predominant variant from October to December 2021, until it was displaced by Omicron (Figure 2A). By analyzing Brazilian sequences from the same period, we observed a similar pattern of Gamma dominance and an increase in the national COVID-19 case numbers in early 2021 (Figure 2B). This wave was followed by Delta’s replacement, which did not correspond to an increase in detected cases or death rates (Figure 2B, D). Omicron’s subsequent introduction and spread towards the end of 2021 led to an unprecedented surge in COVID-19 cases, accompanied by only a modest increase in deaths (Figure 2A-D).

**Figure 3.**
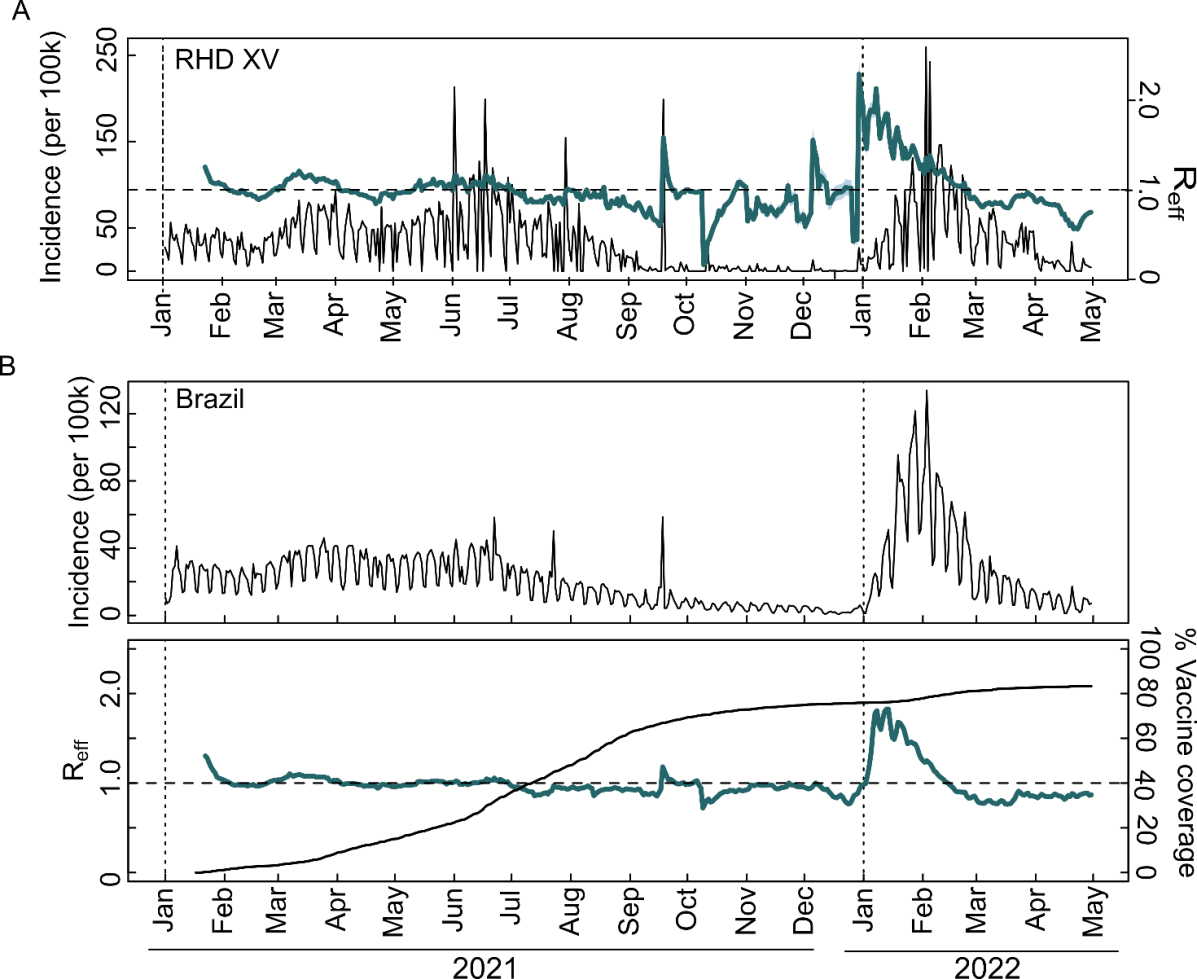
Prevalence and spread of SARS-CoV-2 infections in the 15th Regional Health District and Brazil. A) Incidence of COVID-19 per 100,000 inhabitants in the RHD XV region on the left y-axis together with estimated effective reproductive number (Reff) for all COVID-19 cases in the region, represented as a purple line on the right y-axis; B) COVID-19 incidence per 100,000 inhabitants for all cases across Brazil and estimated effective reproductive number (Reff) over time (purple line) for all reported COVID-19 cases (right y-axis), including the percentage of vaccine coverage in the entire population during the same timeframe (left y-axis).

Interestingly, despite Delta replacing Gamma in October 2021, there was no corresponding increase in case numbers. This scenario changed in December 2021 with the detection of Omicron, leading to a noticeable shift in case trends (Figure 2A, B). Reinforcing these findings, the analysis of effective reproduction number (Reff) revealed peaks (Reff >1) from March to April 2021, late September 2021, and December 2021 to January 2022, indicating a higher transmission rate (Figure 3A and B). This increase in Reff corresponds with the introduction and growth in frequency of the Gamma, Delta, and Omicron VOCs in RHD XV municipalities and across Brazil (Figure 3A and B), suggesting that vaccine effectiveness reduced cases. Notably, even though a large number of cases were reported from January 2022, death only marginally increased. This trend is likely due to the nature of reported cases rather than increased disease severity, contrasting with the period of Gamma dominance and reinforcing the protective effect of vaccination coverage (Figure 2C and D).

**Figure 4.**
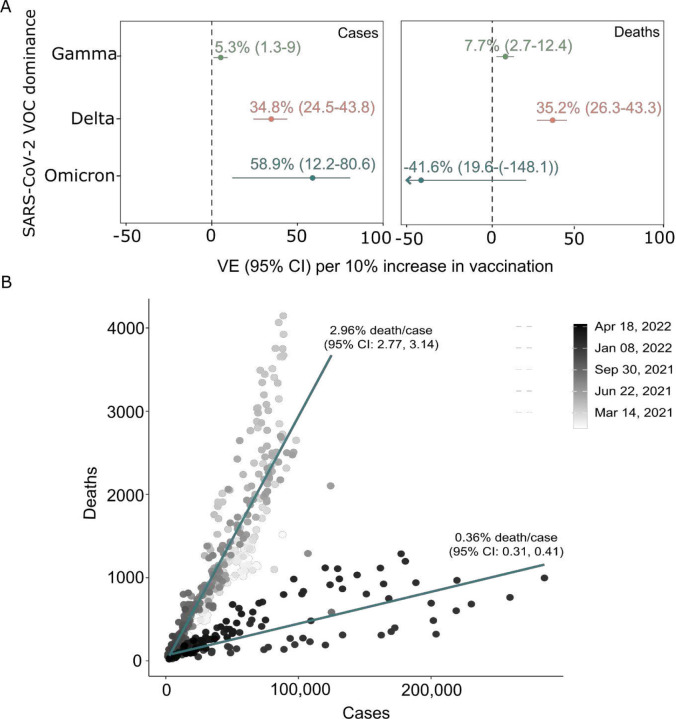
Vaccine effectiveness on the number of COVID-19 cases and deaths. A) Effectiveness of vaccination per 10% increase in vaccination uptake in preventing new COVID-19 cases and deaths in Brazil. B) Number of new deaths per new cases observed in 2021 and 2022.

To further analyze the role of vaccination in Brazil relative to VOC dominance, we looked at the vaccine effectiveness for every 10% increase in overall vaccination uptake in terms of cases and death numbers, adjusting for the number of tests administered (Figure 4A). We found that vaccine effectiveness in preventing cases during the period dominated by Delta and Omicron (1 August - 1 November 2021 and 1 December 2021 – 30 April 2022, respectively) were six and 11 times higher than when Gamma was the dominant variant (1 February - 31 July 2021). Similarly, vaccine effectiveness in preventing deaths during Delta’s dominance was higher than Gamma’s. However, vaccination showed no statistical effect in preventing deaths during Omicron’s dominance, which could be anticipated if the case fatality rate varied over time independent of vaccination. This variation is illustrated in Figure 4B, where the deaths-to-case ratio decreased from 2.96% (95% CI: 2.77, 3.14) before December 2021 to 0.36% (95% CI: 0.31, 0.41) from December 2021 onwards.

### SARS-CoV-2 phylodynamic analysis in the in northwestern São Paulo state region

**Figure 5.**
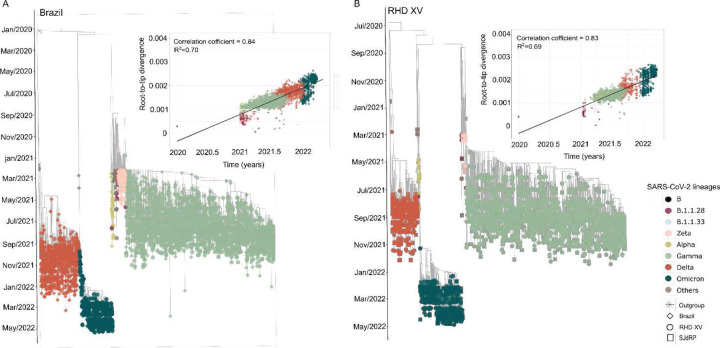
Maximum-likelihood tree for SARS-CoV-2 based on complete genome sequences from the 15^th^ Regional Health District and Brazilian regions. A) Time-stamped phylogenetic tree reconstructed using 4,520 Brazilian complete genomes (3,293 from this study) (Supplementary Table 2) and linear regression of root-to-tip genetic distance of different SARS-CoV-2 lineages versus sampling date. Colors represent different locations. B) Time-stamped phylogenetic tree reconstructed using 3,293 genome sequences from the RHD XV region, including 962 from SJdRP (Supplementary Table 1) and linear regression of root-to-tip genetic distance of different SARS-CoV-2 lineages versus sampling date. Colors represent different locations.

To better understand the dynamics of SARS-CoV-2 lineages and their spread in the study area, we investigated the phylodynamics of the different lineages detected in the RHD XV and the lineages circulating in Brazil during the sampled period. We reconstructed a time-scaled maximum likelihood (ML) tree using 1,227 genomes from all Brazilian states and 3,293 genomes from 85 cities from the RHD XV sequenced in this study (Figure 5A, Supplementary Tables 2). Phylogenetic analysis showed three main clades corresponding to the Gamma, Delta, and Omicron variants and depicted the clade replacement events over time. Importantly, in the different clades, we observed that RHD XV sequences were interspersed with Brazilian sequences from different geographic regions, suggesting several introduction events (Figure 5A).

Similarly, in order to understand the dynamics of SARS-CoV-2 within the region of study, we built a time-scaled ML tree including only sequences from the RHD XV (Supplementary Table 1) and highlighting SJdRP since this municipality had the most significant number of cases, sequenced genomes (Figure 1 A, B), and played an essential role during the COVID-19 pandemic, with one of the most important care centers in the state. In the Brazilian Maximum Likelihood (ML) tree shown in Figure 5A, we observed the clustering of sequences into three distinct groups. These groups correspond to the primary lineages of the virus identified in 2021, as further detailed in Figure 5B. Additionally, the sequences from SJdRP were found distributed across various clades, along with sequences from other cities in the RHD XV region. This pattern, depicted in Figure 5B, suggests multiple introductions of the virus being imported into and exported from these areas.

### Spatiotemporal dispersion of the Gamma, Delta, and Omicron lineages

**Figure 6.**
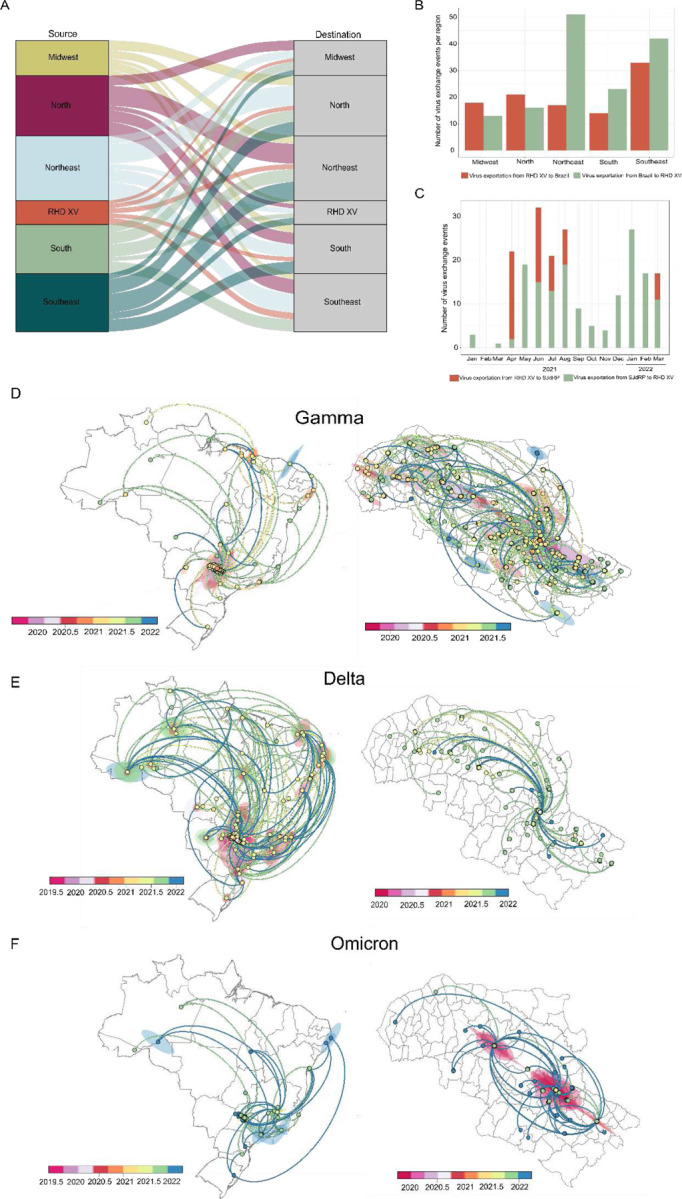
Spatiotemporal spread of Gamma, Delta, and Omicron variants in Brazil and within the 15^th^ Regional Health District (RHD XV). A) Number of all SARS-CoV-2 lineages exchanged between RHD XV municipalities and all Brazilian regions (North, Northeast, Midwest, Southeast, and South) from 2021 and early 2022. B) Source of viral exports and imports of all SARS-CoV-2 lineages identified in 2021 and early 2022 from Brazil to cities in RHD XV and (C) from RHD XV to SJRP. Phylogeographic reconstruction and dispersion of the (D) Gamma variant, (E) Delta variant, and (F) Omicron variant in Brazil and in RHD XV municipalities. Shaded areas represent the 80% highest posterior density interval and depict the uncertainty of the phylogeographic estimates for each node. Curved lines show the links between nodes and viral movement to different locations. Circles represent nodes of the maximum clade credibility phylogeny, and their colors represent the inferred time of occurrence.

Based on our spatiotemporal analyses, we could infer the number of virus exchanges between the RHD XV and the Brazilian regions and between SJdRP and surrounding cities within the RHD XV region. We found that cities in the RHD XV exchanged SARS-CoV-2 variants with all country regions (Figure 6A, Supplementary Table 3). The highest number of imported virus variants in the RHD XV region originated predominantly from Brazil’s northeast and southeast, with at least 51 and 42 importation events, respectively. In contrast, most variants exported from the RHD XV region were sent to the southeast, accounting for at least 33 exportation events, as shown in Figure 6B and detailed in Supplementary Table 3. Analysis of transmission dynamics within the RHD XV showed that SJdRP contributed to virus exchange during 2021 (Figure 6C). Most virus exchange events were exports from SJdRP to other municipalities (at least 157). However, interestingly, most of the importation events (at least 120) took place between April and August 2021, coinciding with the wide circulation of the Gamma variant in the RHD XV region (Figure 6C, Supplementary Table 3).

Next, we investigated the spatiotemporal history of the VOCs involved in the three major clade replacement events and were responsible for at least two significant COVID-19 epidemic waves in Brazil during 2021. Our phylogeographic analyses traced the movement of the Gamma lineage across the different Brazilian regions and its interaction with the RHD XV region. This analysis revealed that the Gamma variant was a prominent presence in Brazil’s epidemiological landscape throughout the period it was prevalent. Additionally, our findings suggest that the initial Gamma sequences identified in the RHD XV region were likely imported from Brazil’s southeastern and northeastern states (Figure 6D). Moreover, we observed a notable migration pattern for the Gamma lineage within the RHD XV region. The evidence suggests that this variant was first introduced in SJdRP and then rapidly spread to other municipalities, with a pronounced spread to neighboring cities (Figure 6D).

The Delta variant, which started to replace the Gamma variant in Brazil and specifically in the RHD XV region by September 2021 (Figure 2A, B), exhibited intense spread across all Brazilian regions. It notably moved between the southeastern, northeastern, and northern states and the RHD XV region (Figure 6E). However, when analyzing virus movement through the RHD XV region, we observed a predominance of exportation events from SJdRP, but to a lesser extent compared to Gamma, which may reflect the lower number of cases identified in that period (Figure 6E). Importation and exportation of Omicron were observed among the RHD XV region and a few states in all Brazilian regions (Figure 6F). As with Delta, Omicron spread mainly from SJdRP to several cities within the RHD XV region (Figure 6C and F); despite the large number of cases reported after the introduction of Omicron, fewer importation and exportation events were observed compared to Gamma, which could suggest that most transmission occurred locally.

## Discussion

In a continent-spanning country like Brazil, limitations on real-time surveillance of SARS-CoV-2 variants have been reported due to different regional sequencing efforts ^19^, which posed a severe challenge to monitor the origin of new mutation events that may enhance virus fitness and persistence of the pandemic at the national level. A possible alternative is to implement genomic surveillance in a small region that exhibits constant virus exchange with several geographic locations and may reflect the progression of the pandemic nationally.

Our findings demonstrated successive clade replacement events, corresponding to the introduction and increased frequency of the SARS-CoV-2 VOCs Gamma, Delta, and Omicron, respectively. This scenario is concerning during a pandemic since subsequent introductions of new lineages may contribute to the resurgence of cases and continuation of the disease, as reported for dengue and influenza ^20–24^. We found that the three SARS-CoV-2 variant replacements in 2021 and early 2022 were linked to two waves of COVID-19, as observed in other studies at local and national levels ^17,19,25–27^. The Gamma lineage emerged in late 2020 in Manaus, the capital of Amazonas state ^9,28^, and it rapidly spread throughout Brazil, replacing Zeta and all other circulating SARS-CoV-2 variants to become the dominant lineage within six months (March–August 2021) across Brazil ^17,19^. According to Naveca et al. 28, the rapid spread of Gamma lineage was associated with the increased travel frequency observed during the Christmas and New Year holidays. Additionally, this VOC’s higher transmissibility rates and immune evasion led to reinfections and breakthrough infections^29–35^. The negative impact of Gamma across Brazil was amplified by the slow vaccination rate of early 2021, its greater severity of the disease, and elevated mortality risk in non-vaccinated patients, factors combined to overload health care systems and leading to a record number of deaths ^17,27^.

Our data also showed that the increased vaccination coverage from July 2021 helped decrease the number of COVID-19 cases and deaths in the RHD XV region as well as in other regions of Brazil. As a result, the spread of Delta was reduced, leading to a significant decrease in the number of cases and deaths at the national level. This finding becomes even more evident considering that previous infection with the Gamma variant itself did not confer a neutralizing antibody titer against the Delta variant as high as the one achieved by vaccination ^36^. These observations contrasted with what was observed internationally, as the emergence of Delta triggered a new epidemic wave in several countries with varying degrees of vaccine coverage, such as India, Indonesia, Thailand, Myanmar, Nepal, several African nations, the United Kingdom, and Israel ^37–39^. This pattern can be associated with the Delta’s ability to evade the immune system, decreased antibody neutralization by mRNA vaccines as well as higher transmissibility ^37,38,40–43^. The most likely reason was the high vaccine coverage in the population when Delta was imported to Brazil (as shown in our study), in addition to immunity acquired from the large number of cases that occurred when the Gamma variant was dominant ^25^. Our Reff analysis demonstrated that when Delta replaced Gamma in September 2021, there was a slight increase in the reproduction number for SARS-CoV-2, quickly followed by a decrease, but this did not impact the number of cases reported at the time, suggesting that higher immune protection of the population helped contain virus spread. Indeed, by the time Delta increased in frequency in the RHD XV, most of the population had been fully vaccinated with two doses of the inactivated-virus Sinovac/CoronaVac vaccines, and booster distribution had begun. Our study shows that these efforts effectively prevented severe cases and deaths. These findings are reinforced by studies demonstrating the effectiveness of the CoronaVac booster in inducing a potent immune response and elevated virus-specific antibody levels, increasing Delta variant neutralization activity, and subsequently preventing infection and severe outcomes ^44–47^

After the introduction and spread of Omicron, we observed a new resurgence of cases (January 2022); however, there was only a slight increase in the number of deaths in municipalities in the RHD XV region, corroborating the epidemiological landscape seen in Brazil as a whole. The Omicron variant was detected in South Africa and Botswana in November 2021, alongside an exponential rise in the incidence of COVID-19 ^11^. Phylogenetic analyses estimated that Omicron emerged in October 2021 with a significant R_0_, contributing to its vast and rapid spread ^11^. Indeed, our analyses showed a sharp peak in the Reff for SARS-CoV-2 (>2) when Omicron was introduced and increased in frequency, which was not observed when Gamma and Delta were the dominant variants, reinforcing the transmissibility of Omicron. The higher transmission rate of this variant is related to the constellation of mutations it displays: over 30 mutations in the spike glycoprotein, several in the receptor binding domain (RBD) and N-terminal domain (NTD), which reduced its sensitivity to neutralization by anti-SARS-CoV-2 antibodies induced by previous infection or vaccination ^11^. Despite Omicron’s pronounced transmissibility compared to Delta ^48^ and higher immune evasion compared to previous VOCs ^49^, it exhibited reduced severity, leading to lower hospitalization rates ^50,51^. Our results showed this same pattern; even though the number of cases rose abruptly from January 2022 in cities in the RHD XV region and throughout Brazil, the number of deaths was much lower than previously observed. The main factor underlying this pattern is likely attributed to the high rates of booster vaccination among the population, which is shown to promote higher titers of neutralizing antibodies ^52–55^ and strong protection against severe disease and death ^56^.

Analyzing the spread of the different VOC in the RDH XV region and Brazil, we observed lower virus exchange among the different Brazilian regions when Gamma was the dominant variant compared to Delta. One explanation is that by the time Gamma was circulating (March–August 2021), Brazil implemented more severe restrictions and social isolation, and vaccination was initiated ^27^. The restrictions had been loosened or abandoned when Delta replaced Gamma, leading to higher virus exchange among the Brazilian states despite the lower numbers of notified COVID-19 cases. Nevertheless, an opposite pattern was observed in RHD XV since a more intense viral movement was reported when Gamma was the prevalent SARS-CoV-2 lineage. This likely resulted from the burst of COVID-19 cases reported from April 2021 that led to a record number of deaths and intensive care unit (ICU) occupancy rates. Because SJdRP has one of the most important centers for COVID-19 care and treatment, the city received people from various municipalities in the RHD XV, leading to higher rates of SARS-CoV-2 importation and exportation; still, when Delta was predominant, the population had been vaccinated full or partially that was fundamental for the decrease of transmission rates. Banho, Sacchetto, et al. ^17^ showed that ICU occupancy reached 100% in SJRP when Gamma was prevalent in the RHD XV, suggesting that the intense virus importations and exportations observed in 2021 were related to SJRP’s role as the headquarters of RHD XV and home to the main hospital responsible for SARS-CoV-2 diagnosis, receiving over 5,700 admissions up to June 2021 ^17^. Nevertheless, during Delta circulation, the vaccine coverage was high, which helped to decrease the severity of the disease, probably leading to lower demand for treatment in more specialized healthcare centers such as HB, and consequently impacting virus circulation and transmission among the cities within the district.

Interestingly, while we expected Omicron to display the same pattern as Delta in Brazil, we observed significantly less virus exchange. This may be related to the sampling coverage nationwide, since from December 2021 to April 2022, only 933 full-coverage complete genomes of SARS-CoV-2 classified as Omicron are available at GISAID (https://www.epicov.org/). The most Brazilian states from which Omicron genomes were retrieved showed poor sampling, which may have influenced the analysis and is a limitation of the study. For the municipalities within the RHD XV region, we observed virus movement among neighboring locations and migration patterns regardless of population density, as seen with the Delta variant. Moreover, it is important to highlight that sampling representativeness differed for Gamma, Delta, and Omicron since SARS-CoV-2 genomes from 84, 41, and 28 municipalities were sampled when Gamma, Delta, and Omicron were the dominant variants, respectively. This difference may limit the analysis of the spatiotemporal spread of SARS-CoV-2 in the RHD XV region. However, at the same time, it suggests that when infected people had milder symptoms (due to vaccine efficiency), fewer sought diagnoses in the health system, thus reducing the availability of collected samples for genomic surveillance.

## Conclusion

Using epidemiological data, genomic sequencing, and phylogeographic analyses, we demonstrated three well-defined clade replacement events in the RHD XV region, corresponding to the introduction and spread of the Gamma, Delta, and Omicron variants. The rapidly increasing prevalence of these VOCs triggered two COVID-19 epidemic waves, which were significantly influenced by the vaccination landscape. Our study revealed that the effectiveness of vaccination in mitigating new cases during Delta and Omicron circulation was six and eleven times higher, respectively, than during Gamma’s dominance. Additionally, vaccination coverage and booster doses were highly effective in reducing cases and deaths at local and national level. Thoroughly, our results revealed that SJdRP played a pivotal role in disseminating SARS-CoV-2 lineages to neighboring cities within the district.. This underscores SJdRP’s suitability as a focal point for genomic surveillance, providing a reliable reflection of the national pattern of SARS-CoV-2 spread and evolution.

## Material and methods

### Ethics Statement

This study was approved by the Ethics Committee of the São José do Rio Preto School of Medicine (FAMERP) (protocol number: CAAE #31588920.0.0000.5415, on November 29, 2021). Written informed consent was waived by the institutional review board (IRB) since all samples were collected for routine diagnosis, and the data were analyzed anonymously, ensuring total confidentiality for all participants.

### Epidemiological data

Data from reported and confirmed COVID-19 cases in Brazil were provided by the Brazilian Ministry of Health and are available at https://github.com/wcota/covid19br ^57^. To perform the analyses, we added the geographical locations (Brazilian regions, namely North, Northeast, Southeast, South, Midwest, and the RHD XV).

The effective reproduction number (Reff) for SARS-CoV-2 over the study period was estimated using the EpiEstim ^58^ in R version 4.3.1 ^59^. We fit the time-varying Reff, assuming a parametric serial interval with a mean of five days using a 21-day sliding window. The Reff reflects the behavior of an epidemic, and by definition is the average number of secondary infections caused by an infected person at a given time, where R > 1 indicates a growing epidemic, while an R < 1 indicates a decrease in transmission. Negative binomial regressions of new cases or deaths by day were run with a 10% increase in the percentage of the vaccinated population, adjusting for the number of new tests for that day. Vaccine effectiveness and 95% confidence intervals (CI) were estimated as 1-exp(coefficient)) or 1-exp(lower or upper CI).

### Clinical samples and molecular investigation

To monitor the epidemiological profile and spatiotemporal dynamics of the SARS-CoV-2 variants that circulated during 2021–2022, convenience samples presenting positive diagnoses for COVID-19 in residents of municipalities within the RHD XV were randomly selected by the SPNPAESV for whole-genome sequencing based on the cycle threshold value (≤30) and availability of epidemiological metadata such as date of sample collection and municipality of residence to perform phylogeographic analyses.

For COVID-19 diagnosis, viral RNA was extracted with the Extracta kit fast DNA and RNA viral (MVXA-P096 FAST; Loccus, Brazil) according to the manufacturer’s instructions, utilizing an Extracta 96 DNA and RNA extractor and purifier (Loccus, Brazil). Reverse transcription followed by real-time polymerase chain reaction (RT-qPCR) was performed with the GeneFinder COVID-19 Plus RealAmp kit (OSANG Healthcare, Korea), targeting the RNA-dependent RNA polymerase (RdRp), envelope (E), and nucleocapsid (N) genes of the SARS-CoV-2 genome and the human RNAse P. The RT-qPCR was conducted in a QuantStudio 5 Real-Time PCR System (Thermo Fisher Scientific, USA), and the results were analyzed in QuantStudio 5 software v1.5.1 (Thermo Fisher Scientific, USA) interpreted as cycle threshold value (Ct) less or equal to 40 as positive..

### Whole-genome sequencing, genome assembling and lineage assignment

Samples presenting Ct value less or equto to 30 were randomly selected for whole-genome sequencing. Whole-genome amplification, and library preparation were performed using Illumina CovidSeq Test (Illumina Inc, USA), according to the instructions provided. The quality and size of the libraries were checked by Agilent 4150 TapeStation (Agilent Technologies Inc, USA). Libraries were pooled in equimolar concentrations, and the sequencing was conducted in the Illumina MiSeq System (Illumina Inc, USA), using MiSeq Reagent Kit v2 (2 × 150 bp cycles) (Illumina Inc, USA). The quality of the raw sequencing data was checked using FastQC software v. 0.11.9 (http://www.bioinformatics.babraham.ac.uk/projects/fastqc) and trimmed with Trimmomatic v. 0.39^62^ to filter low-quality reads, low-quality bases, and reads with at least 75 base pairs (bp). The cleaned paired-end reads were mapped against the Wuhan-Hu-1 reference genome (NC_045512.2) using BWA mem v. 0.7.17 software ^63^ and SAMtools v. 1.10 ^64^ for read sorting and indexing. Next, Pilon software ^65^ was used to improve insertion and deletion detection. Finally, SAMtools v. 1.10 was used to access the position depths in the BAM alignment, and SAMtools mpileup and iVar v. 1.3.1 ^66^were used to generate the consensus genomes (nucleotide positions presenting read depth <10 were considered ‘N’). The generated genomes were subjected to the Pangolin COVID-19 Lineage Assigner Tool version v. 4.0.5 ^67^ to confirm the variant classification.

### Phylogenetic analysis

The datasets used for the phylogenetic analysis included Brazilian SARS-CoV-2 complete genome sequences of collected from January 2021 to April 2022 and the SARS-CoV-2 reference genome retrieved from the GISAID database ^68^. For the Brazilian phylogeny, we used a total of 4,520 whole genomes, and for the RHD XV tree, we used a total of 3,293 whole-genome sequences obtained in this study (Supplementary Tables 1 and 2). All the Brazilian genomes used in this study were downloaded from the GISAID database ^68^, based on the criteria high-coverage and metadata availability. Next, the sequences were selected according to the collection date (from January 2021 to April 2022) and the location (capital cities of each Brazilian state). Nucleotide sequences were aligned using MAFFT v. 7.271 ^69^. Time-scale phylogenetic trees using the maximum-likelihood (ML) method were reconstructed in IQ-TREE v. 2.0.3.7 ^70^, using the best-fit model of nucleotide substitution according to the Bayesian information criterion (BIC) inferred by the ModelFinder tool ^71^. The reliability of branching patterns was tested using a combination of ultrafast bootstrap (UFBoot) and the SH-like approximate likelihood-ratio test (SH-aLRT) ^72^. To investigate the temporal signal from the ML trees, we regressed root-to-tip genetic distances against sample collection dates using the TempEst tool v. 1.5.1 ^73^, considering correlation coefficient >0.4 to accept temporal structure ^74^. Next, generated phylogenies were submitted to TreeTime v. 0.9.3 ^75^ to convert the raw ML trees into time-scaled trees, considering a constant molecular rate of 8.0×10^−4^ nucleotide substitutions per site per year, according to Giovanetti et al. ^27^. Finally, we used the time-scaled tree topologies to infer the number of viral exchange events between the five Brazilian regions and the RHD XV as well as within the RHD XV using TreeTime mugration v. 0.9.3, and by mapping the locations to tips and internal nodes from the annotated tree topology we were able to estimate the number of transition events (virus importations and exportations) among regions/cities ^75^.

### Phylogeography analysis

To better understand the spatiotemporal history of SARS-CoV-2 spread and transmission within the RHD XV and between this district and other regions of Brazil, we investigated the main variants circulating in the region in 2021 and early 2022. To do so, we identified monophyletic clades in the time-scaled phylogenetic trees for the main VOC circulating in 2021 (Gamma, Delta, and Omicron) and randomly extracted sequences from all different clades in each monophyletic group using the Microreact web application ^76^ to infer continuous phylogeography histories using the Markov chain Monte Carlo (MCMC) method in BEAST v1.10.4 software ^77^, as described by Giovanetti et al. ^27^. As a result of this, for each lineage we reconstructed an ML tree and accessed the molecular clock signal using the root-to-tip regression method implemented in TempEst v. 1.5.3 ^73^ and removed outliers that may violate the molecular clock assumption. Next, we down-sampled the lineages to <600 taxa per clade to infer the phylogeography history using BEAST v1.10.4 ^77^ and employing HKY as the nucleotide substitution model, a strict molecular clock, Bayesian skyline model as the coalescent tree prior. We also utilized a flexible relaxed random walk diffusion model ^78,79^ with Cauchy distribution and jitter window site of 0.01 to model the phylogenetic diffusion and spread of each lineage among the Brazilian regions and within the RHD XV. The MCMC chains were run for 250 million interactions and sampled every 25,000 steps. Convergence was assessed in Tracer v. 1.7 ^80^, and maximum clade credibility trees were summarized using Treeannotator v. 1.6.1 after discarding the initial 10% of steps as burn-in. Finally, SERAPHIM ^81^, a package in R software v. 4.2.3^60^, was used to extract and map the spatiotemporal information in the posterior trees.

### Geoprocessing

Databases that included the number of COVID-19 cases per municipality and the number of SARS-CoV-2 genomes sequenced per city were created according to the location/municipality of origin and sample collection date. Maps were created using R software version 3.6.3 ^60^. The shapefiles used in this study are available at: https://www.ibge.gov.br/geociencias/organizacao-do-territorio/malhas-territoriais/15774-malhas.html^61^.

## Supplementary Material

Supplement 1

## Data Availability

All SARS-CoV-2 genomes generated and analyzed in this study are available in the EpiCoV database in GISAID^68^, and their respective access numbers are provided in the Supplementary Material.

## References

[1] Duarte C. M. Rapid evolution of SARS-CoV-2 challenges human defenses. Sci Rep 12, (2022). 10.1038/s41598-022-10097-z PMC9017738 35440671

[2] Markov P. V. The evolution of SARS-CoV-2. Nature Reviews Microbiology vol. 21 Preprint at 10.1038/s41579-023-00878-2 (2023). 37020110

[3] Pybus O. G. & Rambaut A. Evolutionary analysis of the dynamics of viral infectious disease. Nature Reviews Genetics vol. 10 Preprint at 10.1038/nrg2583 (2009). PMC7097015 19564871

[4] Garcia-Beltran W. F. Multiple SARS-CoV-2 variants escape neutralization by vaccine-induced humoral immunity. Cell 184, (2021). 10.1016/j.cell.2021.04.006 PMC8082941 33930298

[5] Chakraborty C., Sharma A. R., Bhattacharya M. & Lee S. S. A Detailed Overview of Immune Escape, Antibody Escape, Partial Vaccine Escape of SARS-CoV-2 and Their Emerging Variants With Escape Mutations. Frontiers in Immunology vol. 13 Preprint at 10.3389/fimmu.2022.801522 (2022). PMC8863680 35222380

[6] Willett B. J. SARS-CoV-2 Omicron is an immune escape variant with an altered cell entry pathway. Nat Microbiol 7, (2022). 10.1038/s41564-022-01143-7 PMC9352574 35798890

[7] Andrew R. Preliminary genomic characterisation of an emergent SARS-CoV-2 lineage in the UK defined by a novel set of spike mutations. Virological.Org (2020).

[8] Zhou D. Evidence of escape of SARS-CoV-2 variant B.1.351 from natural and vaccine-induced sera. Cell 184, (2021). 10.1016/j.cell.2021.02.037 PMC7901269 33730597

[9] Faria N. R. Genomics and epidemiology of the P.1 SARS-CoV-2 lineage in Manaus, Brazil. Science (1979) 372, (2021). 10.1126/science.abh2644 PMC8139423 33853970

[10] Wall E. C. Neutralising antibody activity against SARS-CoV-2 VOCs B.1.617.2 and B.1.351 by BNT162b2 vaccination. The Lancet vol. 397 Preprint at 10.1016/S0140-6736(21)01290-3 (2021). PMC8175044 34090624

[11] Viana R. Rapid epidemic expansion of the SARS-CoV-2 Omicron variant in southern Africa. Nature 603, (2022). 10.1038/s41586-022-04411-y PMC8942855 35042229

[12] Adamoski D. SARS-CoV-2 Delta and Omicron Variants Surge in Curitiba, Southern Brazil, and Its Impact on Overall COVID-19 Lethality. Viruses 14, (2022). 10.3390/v14040809 PMC9027352 35458539

[13] Wolf J. M., Wolf L. M., Bello G. L., Maccari J. G. & Nasi L. A. Molecular evolution of SARS-CoV-2 from December 2019 to August 2022. Journal of Medical Virology vol. 95 Preprint at 10.1002/jmv.28366 (2023). PMC9877913 36458547

[14] Assato P. A. Retrospective Insights of the COVID-19 Epidemic in the Major Latin American City, São Paulo, Southeastern Brazil. Viruses 15, (2023). 10.3390/v15020327 PMC9965911 36851541

[15] Ministério da Saúde. Painel de casos de doença pelo coronavírus 2019 (COVID-19) no Brasil pelo Ministério da Saúde. Access on September 11th, 2023 (2023).

[16] Governo do estado de São Paulo. SP contra o novo coronavirus - Boletim completo. Access on September 11th, 2023 (2023).

[17] Banho C. A. Impact of SARS-CoV-2 Gamma lineage introduction and COVID-19 vaccination on the epidemiological landscape of a Brazilian city. Communications Medicine 2, (2022). 10.1038/s43856-022-00108-5 PMC9053258 35603276

[18] Governo do Estado de São Paulo. Secretaria de Estado da Saúde São Paulo - SES-SP. Access on September 11th, 2023 (2023).

[19] Alcantara L. C. J. SARS-CoV-2 epidemic in Brazil: how the displacement of variants has driven distinct epidemic waves. Virus Res 315, (2022). 10.1016/j.virusres.2022.198785 PMC9022374 35461905

[20] OhAinle M. Dynamics of dengue disease severity determined by the interplay between viral genetics and serotype-specific immunity. Sci Transl Med 3, (2011). 10.1126/scitranslmed.3003084 PMC4517192 22190239

[21] Rico-Hesse R. Origins of dengue type 2 viruses associated with increased pathogenicity in the Americas. Virology 230, (1997). 10.1006/viro.1997.8504 9143280

[22] Lambrechts L. Dengue-1 Virus Clade Replacement in Thailand Associated with Enhanced Mosquito Transmission. J Virol 86, (2012). 10.1128/JVI.06458-11 PMC3264336 22130539

[23] Huang K. Establishment and Lineage Replacement of H6 Influenza Viruses in Domestic Ducks in Southern China. J Virol 86, (2012). 10.1128/JVI.06389-11 PMC3372190 22438558

[24] Drumond B. P., Mondini A., Schmidt D. J., Bosch I. & Nogueira M. L. Population dynamics of DENV-1 genotype V in Brazil is characterized by co-circulation and strain/lineage replacement. Arch Virol 157, (2012). 10.1007/s00705-012-1393-9 22777179

[25] Giovanetti M. Replacement of the Gamma by the Delta variant in Brazil: Impact of lineage displacement on the ongoing pandemic. Virus Evol 8, (2022). 10.1093/ve/veac024 PMC8971541 35371559

[26] Slavov S. N. Dynamics of SARS-CoV-2 Variants of Concern in Vaccination Model City in the State of Sao Paulo, Brazil. Viruses 14, (2022). 10.3390/v14102148 PMC9609010 36298703

[27] Giovanetti M. Genomic epidemiology of the SARS-CoV-2 epidemic in Brazil. Nat Microbiol 7, (2022). 10.1038/s41564-022-01191-z PMC9417986 35982313

[28] Naveca F. G. COVID-19 in Amazonas, Brazil, was driven by the persistence of endemic lineages and P.1 emergence. Nat Med 27, (2021). 10.1038/s41591-021-01378-7 34035535

[29] Romano C. M. Sars-cov-2 reinfection caused by the p.1 lineage in araraquara city, sao paulo state, brazil. Rev Inst Med Trop Sao Paulo 63, (2021). 10.1590/S1678-9946202163036 PMC8075619 33909850

[30] Fabiani M., Margiotti K., Viola A., Mesoraca A. & Giorlandino C. Mild symptomatic sars-cov-2 p.1 (b.1.1.28) infection in a fully vaccinated 83-year-old man. Pathogens 10, (2021). 10.3390/pathogens10050614 PMC8156209 34067881

[31] Estofolete C. F. Case study of two post vaccination SARS-CoV-2 infections with P1 variants in coronavac vaccinees in Brazil. Viruses 13, (2021). 10.3390/v13071237 PMC8309964 34206727

[32] Souza W. M. Neutralisation of SARS-CoV-2 lineage P.1 by antibodies elicited through natural SARS-CoV-2 infection or vaccination with an inactivated SARS-CoV-2 vaccine: an immunological study. Lancet Microbe 2, (2021). 10.1016/S2666-5247(21)00129-4 PMC8266272 34258603

[33] Liu H. The basis of a more contagious 501Y.V1 variant of SARS-CoV-2. Cell Research vol. 31 Preprint at 10.1038/s41422-021-00496-8 (2021). PMC8063779 33893398

[34] Chen R. E. Resistance of SARS-CoV-2 variants to neutralization by monoclonal and serum-derived polyclonal antibodies. Nat Med 27, (2021). 10.1038/s41591-021-01294-w PMC8058618 33664494

[35] Wang Z. mRNA vaccine-elicited antibodies to SARS-CoV-2 and circulating variants. Nature 592, (2021). 10.1038/s41586-021-03324-6 PMC8503938 33567448

[36] Fumagalli M. J. CoronaVac and ChAdOx1 Vaccination and Gamma Infection Elicited Neutralizing Antibodies against the SARS-CoV-2 Delta Variant. Viruses 14, (2022). 10.3390/v14020305 PMC8880081 35215895

[37] Planas D. Reduced sensitivity of SARS-CoV-2 variant Delta to antibody neutralization. Nature 596, (2021). 10.1038/s41586-021-03777-9 34237773

[38] Tian D., Sun Y., Zhou J. & Ye Q. The Global Epidemic of the SARS-CoV-2 Delta Variant, Key Spike Mutations and Immune Escape. Frontiers in Immunology vol. 12 Preprint at 10.3389/fimmu.2021.751778 (2021). PMC8669155 34917076

[39] Dhar M. S. Genomic characterization and epidemiology of an emerging SARS-CoV-2 variant in Delhi, India. Science (1979) 374, (2021). 10.1126/science.abj9932 PMC7612010 34648303

[40] Campbell F. Increased transmissibility and global spread of SARSCoV-2 variants of concern as at June 2021. Eurosurveillance 26, (2021). 10.2807/1560-7917.ES.2021.26.24.2100509 PMC8212592 34142653

[41] Wilhelm A. Antibody-mediated neutralization of authentic sars-cov-2 b.1.617 variants harboring l452r and t478k/e484q. Viruses 13, (2021). 10.3390/v13091693 PMC8473269 34578275

[42] Liu C. Reduced neutralization of SARS-CoV-2 B.1.617 by vaccine and convalescent serum. Cell 184, (2021). 10.1016/j.cell.2021.06.020 PMC8218332 34242578

[43] Davis C. Reduced neutralisation of the Delta (B.1.617.2) SARS-CoV-2 variant of concern following vaccination. PLoS Pathog 17, (2021). 10.1371/journal.ppat.1010022 PMC8639073 34855916

[44] Ranzani O. T. Effectiveness of an inactivated Covid-19 vaccine with homologous and heterologous boosters against Omicron in Brazil. Nat Commun 13, (2022). 10.1038/s41467-022-33169-0 PMC9537178 36202800

[45] Grenfell R. F. Q. Immunogenicity, Effectiveness, and Safety of Inactivated Virus (CoronaVac) Vaccine in a Two-Dose Primary Protocol and BNT162b2 Heterologous Booster in Brazil (Immunita-001): A One Year Period Follow Up Phase 4 Study. Front Immunol 13, (2022). 10.3389/fimmu.2022.918896 PMC9218743 35757764

[46] Cerqueira-Silva T. Vaccine effectiveness of heterologous CoronaVac plus BNT162b2 in Brazil. Nat Med 28, (2022). 10.1038/s41591-022-01701-w PMC9018414 35140406

[47] Pérez-Then E. Neutralizing antibodies against the SARS-CoV-2 Delta and Omicron variants following heterologous CoronaVac plus BNT162b2 booster vaccination. Nat Med 28, (2022). 10.1038/s41591-022-01705-6 PMC8938264 35051990

[48] Burki T. K. Omicron variant and booster COVID-19 vaccines. Lancet Respir Med 10, (2022). 10.1016/S2213-2600(21)00559-2 PMC8683118 34929158

[49] Akkız H. The Biological Functions and Clinical Significance of SARS-CoV-2 Variants of Corcern. Frontiers in Medicine vol. 9 Preprint at 10.3389/fmed.2022.849217 (2022). PMC9163346 35669924

[50] Butt A. A. Coronavirus Disease 2019 Disease Severity in Children Infected With the Omicron Variant. Clinical Infectious Diseases 75, (2022). 10.1093/cid/ciac275 PMC9047187 35404391

[51] Menni C. Symptom prevalence, duration, and risk of hospital admission in individuals infected with SARS-CoV-2 during periods of omicron and delta variant dominance: a prospective observational study from the ZOE COVID Study. The Lancet 399, (2022). 10.1016/S0140-6736(22)00327-0 PMC8989396 35397851

[52] Andrews N. Covid-19 Vaccine Effectiveness against the Omicron (B.1.1.529) Variant. New England Journal of Medicine 386, (2022). 10.1056/NEJMoa2119451 PMC8908811 35249272

[53] Magen O. Fourth Dose of BNT162b2 mRNA Covid-19 Vaccine in a Nationwide Setting. New England Journal of Medicine 386, (2022). 10.1056/NEJMoa2201688 PMC9020581 35417631

[54] Regev-Yochay G. Efficacy of a Fourth Dose of Covid-19 mRNA Vaccine against Omicron. New England Journal of Medicine 386, (2022). 10.1056/NEJMc2202542 PMC9006792 35297591

[55] Chansaenroj J. Immunogenicity Following Two Doses of the BBIBP-CorV Vaccine and a Third Booster Dose with a Viral Vector and mRNA COVID-19 Vaccines against Delta and Omicron Variants in Prime Immunized Adults with Two Doses of the BBIBP-CorV Vaccine. Vaccines (Basel) 10, (2022). 10.3390/vaccines10071071 PMC9317843 35891235

[56] Nyberg T. Comparative analysis of the risks of hospitalisation and death associated with SARS-CoV-2 omicron (B.1.1.529) and delta (B.1.617.2) variants in England: a cohort study. The Lancet 399, (2022). 10.1016/S0140-6736(22)00462-7 PMC8926413 35305296

[57] Cota W., Rodrigues C. & Bil L. Monitoring the number of COVID-19 cases and deaths in Brazil at municipal and federative units level. SciELO Preprints (2020).

[58] Cori A., Ferguson N. M., Fraser C. & Cauchemez S. A new framework and software to estimate time-varying reproduction numbers during epidemics. Am J Epidemiol 178, (2013). 10.1093/aje/kwt133 PMC3816335 24043437

[59] R Core Team. R: A language and environment for statistical computing. Preprint at (2023).

[60] R Core Team. R: A language and environment for statistical computing.. Preprint at (2020).

[61] Instituto Brasileiro de Geografia e Estatística. Malha Municipal. Access on September 12, 2023 (2023).

[62] Bolger A. M., Lohse M. & Usadel B. Trimmomatic: A flexible trimmer for Illumina sequence data. Bioinformatics 30, (2014). 10.1093/bioinformatics/btu170 PMC4103590 24695404

[63] Li H. & Durbin R. Fast and accurate short read alignment with Burrows-Wheeler transform. Bioinformatics 25, (2009). 10.1093/bioinformatics/btp324 PMC2705234 19451168

[64] Li H. The Sequence Alignment/Map format and SAMtools. Bioinformatics 25, (2009). 10.1093/bioinformatics/btp352 PMC2723002 19505943

[65] Walker B. J. Pilon: An integrated tool for comprehensive microbial variant detection and genome assembly improvement. PLoS One 9, (2014). 10.1371/journal.pone.0112963 PMC4237348 25409509

[66] Grubaugh N. D. An amplicon-based sequencing framework for accurately measuring intrahost virus diversity using PrimalSeq and iVar. Genome Biol 20, (2019). 10.1186/s13059-018-1618-7 PMC6325816 30621750

[67] O’Toole Á. Assignment of epidemiological lineages in an emerging pandemic using the pangolin tool. Virus Evol 7, (2021). 10.1093/ve/veab064 PMC8344591 34527285

[68] Elbe S. & Buckland-Merrett G. Data, disease and diplomacy: GISAID’s innovative contribution to global health. Global Challenges 1, (2017). 10.1002/gch2.1018 PMC6607375 31565258

[69] Katoh K. & Standley D. M. MAFFT multiple sequence alignment software version 7: Improvements in performance and usability. Mol Biol Evol 30, (2013). 10.1093/molbev/mst010 PMC3603318 23329690

[70] Nguyen L. T., Schmidt H. A., Von Haeseler A. & Minh B. Q. IQ-TREE: A fast and effective stochastic algorithm for estimating maximum-likelihood phylogenies. Mol Biol Evol 32, (2015). 10.1093/molbev/msu300 PMC4271533 25371430

[71] Kalyaanamoorthy S., Minh B. Q., Wong T. K. F., Von Haeseler A. & Jermiin L. S. ModelFinder: Fast model selection for accurate phylogenetic estimates. Nat Methods 14, (2017). 10.1038/nmeth.4285 PMC5453245 28481363

[72] Anisimova M. & Gascuel O. Approximate likelihood-ratio test for branches: A fast, accurate, and powerful alternative. Syst Biol 55, (2006). 10.1080/10635150600755453 16785212

[73] Rambaut A., Lam T. T., Carvalho L. M. & Pybus O. G. Exploring the temporal structure of heterochronous sequences using TempEst (formerly Path-O-Gen). Virus Evol 2, (2016). 10.1093/ve/vew007 PMC4989882 27774300

[74] Tegally H. Sixteen novel lineages of SARS-CoV-2 in South Africa. Nat Med 27, (2021). 10.1038/s41591-021-01255-3 33531709

[75] Sagulenko P., Puller V. & Neher R. A. TreeTime: Maximum-likelihood phylodynamic analysis. Virus Evol 4, (2018). 10.1093/ve/vex042 PMC5758920 29340210

[76] Argimón S. Microreact: visualizing and sharing data for genomic epidemiology and phylogeography. Microb Genom 2, (2016). 10.1099/mgen.0.000093 PMC5320705 28348833

[77] Suchard M. A. Bayesian phylogenetic and phylodynamic data integration using BEAST 1.10. Virus Evol 4, (2018). 10.1093/ve/vey016 PMC6007674 29942656

[78] Dellicour S. Relax, Keep Walking - A Practical Guide to Continuous Phylogeographic Inference with BEAST. Mol Biol Evol 38, (2021). 10.1093/molbev/msab031 PMC8321535 33528560

[79] Lemey P., Rambaut A., Welch J. J. & Suchard M. A. Phylogeography takes a relaxed random walk in continuous space and time. Mol Biol Evol 27, (2010). 10.1093/molbev/msq067 PMC2915639 20203288

[80] Rambaut A., Drummond A. J., Xie D., Baele G. & Suchard M. A. Posterior summarization in Bayesian phylogenetics using Tracer 1.7. Syst Biol 67, (2018). 10.1093/sysbio/syy032 PMC6101584 29718447

[81] Dellicour S., Rose R., Faria N. R., Lemey P. & Pybus O. G. SERAPHIM: Studying environmental rasters and phylogenetically informed movements. Bioinformatics 32, (2016). 10.1093/bioinformatics/btw384 27334476

