# Supplementary material for: Dynamic clade transitions and the influence of vaccine rollout on the spatiotemporal circulation of SARS-CoV-2 variants in São Paulo, Brazil
Source: Res Sq. 2024 Jan 22:rs.3.rs-3788142. Preprint. [Version 1] doi: 10.21203/rs.3.rs-3788142/v1 (PMC10854302; doi:10.21203/rs.3.rs-3788142/v1)
Supplement: Supplement 1 [file NIHPPRS3788142v1-supplement-1.pdf]

Supplementary Files

This is a list of supplementary files associated with this preprint. Click to download.

- SupplementaryTable13.xlsx
